# Patient severity matters for night-shift workload for internal medicine residents in Taiwan

**DOI:** 10.1186/s12913-014-0587-0

**Published:** 2014-12-03

**Authors:** Nin-Chieh Hsu, Ming-Chin Yang, Ray-E Chang, Wen-Je Ko

**Affiliations:** Division of Hospital Medicine, Department of Traumatology, National Taiwan University Hospital, Taipei, Taiwan; Department of Internal Medicine, National Taiwan University Hospital, Taipei, Taiwan; Institute of Health Policy and Management, College of Public Health, National Taiwan University, Rm.639, #17 Xu-Zhou Road, Taipei, Taiwan

**Keywords:** After-hours care, Workload, Resident, Hospitalist

## Abstract

**Background:**

Although work hour is an important factors for resident workload, other contributing factors, such as patient severity, with regards to resident workload have been scarcely studied.

**Methods:**

A prospective observational cohort study was conducted in a general medicine unit in an academic medical center in Taiwan. Every event for which the nurses needed to call the on-call residents was recorded. To quantify the workload, the responses of on-duty residents to calls were analyzed. To allow comparisons of patient factors to be made, we classified all patients by assigning them stable, unstable, or do-not-resuscitate (DNR) codes. The reasons for the calls were categorized to facilitate the comparisons across these three groups.

**Results:**

From October 2009 to September 2011, a total of 2,518 patients were admitted to the general medicine unit. The nurses recorded a total of 847 calls from 730 call nights, ranging from 0 to 7 per night. Two peaks of calls, at 0-2 am and 6-7 am, were noted. Calls from stable, unstable, and DNR patients were 442 (52.2%), 95 (11.2%), and 298 (35.2%), respectively. For both unstable and DNR patients, the leading reason was abnormal vital signs (62.1% and 67.1%, respectively), while only 36.2% for stable patients. Both unstable and DNR patients required more bedside evaluation and management compared to stable patients.

**Conclusion:**

Beyond work hours and patient census, patients with different clinical severity and palliative goal produce different workload for on-call residents.

## Background

Shift work, which improves inpatient care during after-hours and on weekends, is an essential component of current inpatient care models [1]. The main workload during on-call shifts includes caring for previously admitted patients and managing new admissions, if any [2]. However, criticism of shift work has been ongoing for decades, mostly as a result of the sleep deprivation and the impaired neuropsychological performance of the workers, which raised concerns about patient safety [3].

The Accreditation Council for Graduate Medical Education (ACGME) in the United States mandated limits on resident work hours in 2003, including a 30-hour limit on continuous shifts [4]. Most controversy focused only on the positive and negative effects of work hour restrictions [5,6], until the conceptual framework of work intensity was emphasized again recently by Horner and coworkers [7]. In Horner’s framework, patient factors, provider factors and practice-based factors were three essential confounders for clinical work demand, which in turn contributed to physician work intensity and influenced physician health and patient outcomes. Work hours, which could be classified as practice factors in the Horner’s model, is one of the contributing factors of workload which has been investigated quite often. However, provider factors and patient factors were relatively neglected in the current literature.

To improve residents’ health, it is reasonable to reduce the night workload as much as possible, unless this could compromise patient safety or quality of care [8]. Libby and coworkers investigated the importance rating of beeper calls to interns, and revealed that nearly 60% of the calls were not relevant to patient care and 37% interrupted teaching or patient-physician interactions [9]. Some researchers also revealed that nurses and doctors exhibited different patterns of paging [10]. Some authors therefore advocated that unnecessary calls at night should be reduced. In addition, paging frequently interrupted work and rest at night [11]. However, few studies have attempted to predict night workload and to devise ways to achieve reasonable on-call workloads. The reasons of calling on-duty residents have been extensively studied in several different settings, but the sources of calls which could be helpful in predicting workload were rarely mentioned. Calls from stable, critically ill patients, or patients in other special conditions, may led to different pattern of care.

In order to contribute to the groundwork of establishing reasonable on-call workload, our research aims to study the night-shift workload for residents and focuses on not only the reasons of placing calls at night, but also the patient sources of calls and workload produced after the calls. Although workload is usually measured by census of patient encounter, we hypothesized that resident workload is associated with patient severity. By comparing the reasons of calls and workload produced after calls, our hypothesis that different patient produced different resident workload could be tested.

## Methods

### Study setting

The study was conducted at the National Taiwan University Hospital (NTUH), a 2,000-bed, acute care, university-affiliated tertiary referral medical center in northern Taiwan. A hospitalist acute general medicine ward was set up in October 2009, with three attending physicians and eight nurse practitioners, to admit general medicine patients from the emergency department [12]. Hospitalists, who had general internal medicine background, served as the in-charge attending physicians for hospitalized patients. The study was based on a longitudinal hospitalist research which was approved by the Research Ethical Committee of NTUH (registration number: 201112161RIC).

Three shifts were designed for our hospitalist system in 2009 and remained unchanged during the study period. The day shift, which started at 8 am and ended at 5 pm, handled 36 beds before the bridge hospitalist came. After that, the day shift person continued handling 18 beds, and handed the other 18 beds over to the bridge person. The bridge shift started at 1 pm and ended at 11 pm, handling initially 18 beds and subsequently, after the day person signed off, 36 beds. New admissions from the emergency department (ED), which typically presented between 11 am and 5 pm, were assigned to both the day shift and the bridge hospitalists. Because numerous ED patients were awaiting admission, all available beds would be fully occupied in the evening, and night shift admissions were rare. The night shift was from 11 pm to 8 am the next morning, taking handoffs from the bridge shift, and covered 36 beds overnight. Nurse practitioners were assigned the day and bridge shift hospitalists, while residents were assigned the night shift hospitalist. Residents were the first to receive calls from the night shift nurses, and they were supervised by a night shift hospitalist. In our hospitalist program, the resident’s workload was restricted to 24 hours per shift, with at least an 18-hour rest period after an on-call shift. Each hospitalist, resident, and nurse practitioner had a low-power mobile phone for communicating with each other.

### Study design

From the beginning of the hospitalist ward, a standard night shift event record form was designed. The night shift nurses, who worked from 11 pm to 8 am, were responsible for recording every event that required calling the on-duty residents. The record sheets were handed to the day shift hospitalists the following day. To avoid observation effect, the on-call residents were blinded to the whole study.

### Measurements

The prospective data collection was analyzed in our study. The night shift record form included the time of the call, the categorized clinical state of the patient, the categorized call reason, vital signs at the time when the call was placed, subsequent evaluation and management by the resident, and the nurses’ satisfaction with the whole management process.

Because the patient severity or acuity changed throughout the hospitalized course, it should be measured at the time when the call was placed. To test our hypothesis that workload is associated with patient severity, patients were classified as do-not-resuscitate (DNR) and non-DNR. Non-DNR patients were further labeled as stable and unstable. Non-DNR patients who met the criteria of clinical alert signs were classified as “unstable”, and nurses knew that they should immediately inform residents or attending physicians. The clinical alert sign system of NTUH included 10 items, which have been published in the literature [13]. The red color would be shown on the electronic health information system (HIS) until the warning signs disappeared. Patients with DNR consents were classified as “DNR”, with a green color on the HIS. The remaining patients had no colors presented in association with their status, and were classified as “stable” in our study. The three-colored patient classification system remained unchanged during the study period.

In order to analyze the workload, the response of the residents was classified as a telephone order, or an immediate or delayed bedside evaluation and management. According to regulations in our hospital, a telephone order had to be repeated, confirmed, and written down by nurses, and residents had to follow the patient and complete standard orders from residents within their shift. Residents could fill prescriptions through the electronic system, and they did not have to go to the nurses’ station for prescribing simple medications, such as sleeping pills and antipyretics. When evaluation or management was necessary, the on-call resident decided whether an immediate (within 15 minutes) bedside visit was required. A bedside visit that occurred over 15 minutes after a call had been placed was defined as a delayed bedside visit [14]. Nurses recorded the actual time lag between the call and the visit. In our study, direct patient care workload was defined as bedside visits by the on-call residents.

The reasons for placing a call were classified into six categories by night shift nurses: (1) abnormal vital signs, (2) original symptom/problem, (3) new-onset symptom/problem, (4) need for physician’s evaluation, prescription, or procedure, (5) need for explanation/communication, and (6) others. The former categories had priority over the later ones. For instance, if a new symptom was associated with abnormal vital signs, such as fever or tachycardia, the call was classified as being placed for an abnormal vital sign. Mild, asymptomatic hypoglycemia required a physician’s evaluation, but symptomatic hypoglycemia was coded as a new-onset symptom/problem. Requests for sleeping pills or painkillers were coded as a need for physician’s evaluation, prescription, or procedure. To address the problems opened by the possibility of multiple choices, the daytime attending physician confirmed the nurses’ record sheets the next day.

The night shift nurses who participated in our study were requested to complete an informed consent process by the institutional review board of NTUH, and their satisfaction with on-duty resident’s management was measured using a Likert scale that included five level of satisfaction: very satisfied, satisfied, unsure, dissatisfied, and very dissatisfied.

The categorization and definition of the study parameters are shown in Table 1.

**Table 1:** **Classification of call reasons with definitions and examples**

**Call reason category**	**Definition**	**Example**
Abnormal vital signs	Abnormal blood pressure, heart rate, respiratory rate, body temperature, oxygen saturation, or consciousness	Hypotension
		Arrhythmia
		Fever or hypothermia
Original symptom/problem	An existing symptom or problem which has been handed over from the previous shift	Cancer pain breakthrough
		Ileus with refractory vomiting
New-onset symptom/problem	A new symptom or problem that was not noticed in the previous shift	Chest pain
		Shortness of breath
		Oliguria
Need for physician’s evaluation, prescription, or procedure	Events that nurses think the physician should evaluate, prescribing orders, or performing medical procedures	Hyperglycemia
		Difficulty in sleeping
		Foley obstruction
Need for explanation or communication	Situations in which the nurses think the physician should answer questions or say something to the patients or relatives	Refusing protective constraints
		Refusing treatment advice
		Angry patient or relative
Others	The physician should be informed but no need for direct evaluation	Falling without obvious injury

### Statistical analysis

The data were analyzed using the SPSS software (version 16, SPSS Inc., Chicago, IL, USA). We compared the basic demographic data, the reasons for night calls, residents’ responses, the time until bedside visits, and nurses’ satisfaction scores in the stable, unstable, and DNR patient groups. Inter-group differences were compared using the Pearson Chi-square test for dichotomous and categorical variables, and the one-way ANOVA test for continuous variables.

## Results

### Night shift calls

From October 2009 to September 2011, a total of 2518 patients were admitted to the hospitalist ward. Table 2 depicts the demographic data of all patients. Within 2 years, a total of 847 night shift calls were recorded by 16 nurses in 730 call nights. The number of call ranged from 0 to 7 per night. Table 3 comparatively presents the characteristics of the calls.

**Table 2:** **Demographics of the study population**

	**All patients (n = 2518)**	
	**n (%)**	**Mean (SD)**
**Age (yr)**		69.1 (15.3)
**Male**	1337 (53.1)	
**Hospital LOS (days)**		9.9 (9.0)
**ICU admission**	99 (3.9)	
**Hospital mortality**	176 (7.0)	
**Diagnosis**		
**Pneumonia**	701 (27.8)	
**COPD**	125 (5.0)	
**CHF**	85 (3.4)	
**Gastrointestinal bleeding**	264 (10.5)	
**IAI**	206 (8.2)	
**Cellulitis**	126 (5.0)	
**UTI**	705 (29.0)	
**Other**	306 (12.2)	
**DNR**	457 (18.1)	
**Disposition**		
**Home**	1834 (72.8)	
**Nursing home**	131 (5.0)	
**Death**	176 (7.0)	
**GHTD**	40 (1.6)	
**Other department/institution**	337 (13.4)	

Data are expressed as mean ± standard deviation or number of cases (%).

*Abbreviations*: *BMI* body mass index, *CCI* Charlson comorbidity index, *CHF* congestive heart failure, *COPD* chronic obstructive pulmonary disease, *DNR* do-not-resuscitate, *GHTD* go home to die, *IAI* intra-abdominal infection, *ICU* intensive care unit, *LOS* length of stay, *UTI* urinary tract infection.

**Table 3:** **Comparison of the reasons the calls were placed and residents’ responses at night in patients with different clinical codes**

	**Stable code (n = 442)**	**Unstable code (n = 95)**	**DNR code (n = 298)**	**P value**
**Demographics**				
Age (yr)	68.5 ± 15.5	72.6 ± 12.8	76.4 ± 14.2	<0.001^a^
Male	51.7%	69.5%	52.0%	0.005^b^
**Classification of call reason**				<0.001^b^
Abnormal vital sign	160 (36.2)	59 (62.1)	200 (67.1)	
Original symptom/problem	47 (10.6)	13 (13.7)	23 (7.7)	
New-onset symptom/problem	109 (24.7)	4 (4.2)	23 (7.7)	
Need for physician’s evaluation, Prescription, or procedure	109 (24.7)	16 (16.8)	43 (14.4)	
Need for explanation or communication	8 (1.8)	2 (2.1)	7 (2.3)	
Others	9 (2.0)	1 (1.1)	2 (0.7)	
**Resident’s response (n = 670)**	(n = 346)	(n = 85)	(n = 228)	<0.001^b^
Immediate visit within 15 minutes	82 (23.7)	30 (35.3)	74 (32.5)	
Telephone order with delayed visit	35 (10.1)	13 (15.3)	54 (23.7)	
Telephone order without visit	229 (66.2)	42 (49.4)	100 (43.9)	
**Nurses’ satisfaction score**	(n =434)	(n =92)	(n =293)	0.147^b^
1	195 (44.9)	51 (55.4)	136 (46.4)	
2	167 (38.5)	33 (35.9)	101 (34.5)	
3	66 (15.2)	6 (6.5)	51(17.4)	
4	4 (0.9)	0 (0)	3 (1.0)	
5	2 (0.5)	2 (2.2)	2 (0.7)	

Data are expressed as mean ± standard deviation or number of cases (%).

*Abbreviations*: *DNR* do-not-resuscitate.

^a^One way ANOVA.

^b^Pearson Chi-Square test.

Stable, unstable, and DNR patients accounted for 442 (52.2%), 95 (11.2%), and 298 (35.2%), respectively, of all the calls. For both unstable and DNR patients, the leading reason for placing the calls was abnormal vital signs (62.1% and 67.1%, respectively). For stable patients, the reasons for the calls were relatively balanced among abnormal vital signs (36.2%), new-onset symptoms or problems (24.7%), and the need for an evaluation, prescription, or procedure (24.7%). The call reasons were statistically different by Pearson Chi-square test (p < 0.001).

**Figure 1:**
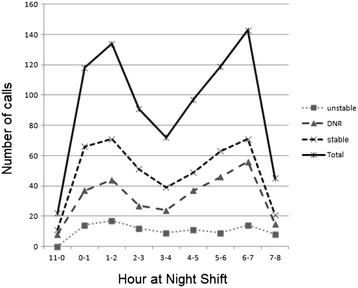
**Time distribution of night shift calls.**

Figure 1 depicts the distribution of 841 calls (the time records were missing for 6 of the calls) on an hourly basis throughout the night shift. Two peaks, at 0-2 am and 6-7 am, were noted. The variation was more prominent for stable and DNR codes than for the unstable code, for which the rate of the calls remained almost constant throughout the night shift.

### On-call resident’s responses

Forty-five residents were observed, and their responses were recorded on 670 (79.7%) forms. The percentage of bedside visits was 33.8%, 50.6%, and 56.1%, and that of immediate (within 15 minutes) visits was 23.7%, 35.3%, and 32.5% after calls received from stable, unstable, and DNR patients, respectively (p < 0.001 by Pearson Chi-square test for immediate visit, delayed visit, and no visit). Regarding the direct patient care workload of on-duty residents, from 289 bedside visits, 40.5%, 14.9%, and 44.3% were from stable, unstable, and DNR patients, respectively.

A total of 819 satisfaction reports from nurses were available for analysis (data were missing for 22 calls). The nurses were “very satisfied” and “satisfied” in 46.6% and 36.8%, of these, respectively, and their level of satisfaction was slightly higher for unstable patients (91.3%) than for stable (83.4%) and DNR ones (80.9%), but did not reach statistical significance (p = 0.147).

Residents’ responses to calls depended on the situation of calls and were therefore complex. Although patients had been labeled as “unstable” in the beginning of the night shift, the nurses might call the resident just to clarify an order or request a sleep pill. It was shown that only 62.1% of calls from unstable patients were due to abnormal vital signs, which may explain why only 50.6% calls required bedside visit by on-call residents. Compared to “stable” patients who only required 33.8% bedside visits by residents, patients labeled as “unstable” produced higher workload.

## Discussion

We believe that our study is the first report about the relationship between patient factors and resident workload. In the conceptual model proposed by Horner, patient factors, provider factors and practice factors contributed to the clinical work demands and work intensity, which in turn influences patient outcomes [7]. Most previous studies addressed the practice factors, such as duty hour design and protected sleep time for residents [15]. However, clinical severity of patient, which is an important patient factor in Horner’s framework, has been scarcely taken into consideration on workload studies.

In the pager era, several ground work studies have already been conducted. Previous studies have revealed the fact that half of pager calls were not relevant to patient care, and most of them did not affect immediate patient management [9,10]. Beebe also conducted important work that investigated the reasons for beeper calls and rated their urgency. Two important findings were that the most common reason for calls (29%) was a change in the patient’s status, and that ratings of urgency made by nurses were not good predictors of the physicians’ responses to the call [14]. However, both studies included complex patient populations, including ward and ICU patients in the former, and pediatric, medical, surgical, and orthopedic patients in the latter. The work done by Katz et al. revealed that nurses and doctors exhibited different patterns of paging medical interns and highlighted unnecessary pages [10]. The study included only medical patients, however it had the limitations of covering relatively short time periods and performing in 1980s. Besides, none of the previous studies attempted to identify and compare the reasons for the calls among different patient groups.

We investigated the sources and reasons of the after-hours calls, their patterns, and the on-call workload in an acute-care general medicine population, and we used a sufficiently long study period. The most valuable finding in our study is that the reasons of night-shift calls were significantly different in patients from different clinical status categories. The workload produced after calls and the behavior pattern of the on-duty residents were also different. In the future, we may predict on-call workload by analyzing the clinical severity status of responsible patients.

Consistent with previous studies that examined night calls, the assessment of patients with complaints or changes in vital signs was the most common reason for the calls in Harvey’s [11] and Wong’s reports [16]. The former used numeric pagers while the latter used alphanumeric pagers that could display both numbers and text. In our study, abnormal vital signs were the leading reason of call in stable, unstable and DNR patients, although the proportion of call reasons was different. These results have important implications for the response to clinical events. There is an inevitable time lag between finding an abnormal patient condition and reaching the on-call physician. Calling via mobile phones may shorten the time lag compared to traditional paging systems, but has been criticized by residents as being highly disruptive for the patient care activity [17]. It calls for more study to prove the advantage of using mobile phones in clinical settings. Besides, most previous studies revealed a low percentage of bedside visits, from 10.3% to 28%, by on-call physicians [11,13]. In our study, immediate (within 15 minutes) bedside visits occurred in 27.8%, and delayed visits in 14.5% of the calls. The higher rate of bedside visits was probably due to a higher proportion of abnormal vital signs in the study population, a different communication style using mobile phones, or a changed doctor-patient relationship and culture that exist nowadays. All patients in this study suffered from acute illness and were admitted from ED, and the picture was typical for an acute general medicine unit. Our study had no historical control and, thus, it could not make further deductions.

Table 4 summarizes the key studies from the related literature that examined pages and calls placed to physicians. In previous studies, the observation periods ranged from 1 week to 18 months. Our study involves the longest study period in the literature to date. A two-year long study period allowed us to minimize the observation effect on the study participants (night shift nurses) and depicted the real practice pattern at night. A recent trial with a comparably long study period investigated the workload of on-call medical interns for 2 years, but paging was not taken into consideration [6]. Our study provided supplemental information to the researchers.

**Table 4:** **Summary of the key studies on pages and calls placed to residents**

**Author, year (reference)**	**Observation period**	**Setting and participants**	**Sample size (tool)**	**Endpoint**	**Important results**
Libby et al. [9]	56 days	Medical service	564 calls (pager)	Importance of call	Nearly 60% of the calls were not relevant to patient care.
		13 interns			The majority of beeper calls do not affect immediate patient management.
Katz et al. [10]	91 days	Internal medicine service in 3 teaching hospitals	1206 calls (pager)	Urgency and reasons of pages, and activities interrupted by pages	65% of the pages interrupted patient care.
		39 interns			Reducing the number of unnecessary pages and postponing nonurgent ones could result in as much as a 42% decrease in the disruption of patient care.
Harvey et al. [11]	1 week	2 teaching hospitals	309 calls (pager)	Number and nature of calls	The most common reasons were prescribing medications (42%), direct patient assessment (25%), and reporting of laboratory results (18%).
		10 interns			Paging frequently interrupts work and rest at night.
Beebe et al. [14]	4 months	Multiple services in one children hospital Nurses	849 calls (pager)	Urgency rating of calls	Nurses’ ratings of the urgency of calls are not good predictors of physician response.
Wong et al. [16]	6 weeks	GIM service	6392 calls^$^	Proportion of text pages, sources of page, page disruption, satisfaction	52% were text pages.
		All health staff	(alpha-numeric pager)		93% of the pages among physicians were text pages.
					There was a 29% reduction in disruptive pages sent during educational rounds.
Patel et al. [19]	18 months	General surgery service	9843 calls (pager)	Sender type, message type, Page quality	As pager volume increased, there was a decrease in the number of pages received per patient.
		6 interns			At higher patient volumes, there was a trend toward an increasing percentage of urgent pages per patient.

^$^There were 1431, 3692 and 1269 pages before, during and after implementation, respectively.

*Abbreviations*: *GIM* general internal medicine.

Our study may have several meaningful clinical implications. First, the 457 DNR patients, who within the study period in our hospitalist setting were a minority (18.1%) of our patient population, surprisingly, accounted for one third of the night calls and nearly one half of the immediate bedside visits made by on-call residents. DNR patients had different reasons for placing the calls as compared to general patients, but had similar reasons as compared to the unstable patients. This finding should be emphasized, because we tend to ignore the workload of palliative care, or so-called conservative care, which is provided to DNR patients. DNR orders for old, co-morbid patients are not uncommon in Taiwan, especially when these patients face acute illness. Our study showed that the workload involved in palliative care was much heavier than expected.

Second, the workload that is required for unstable patients was distributed evenly throughout the whole night. Similar results were found in Beebe’s work, where the number of emergency calls was comparable regardless of whether they occurred during the day or the night [14]. In contrast to the stable and DNR patients, the conditions affecting unstable patients happened at any time, and often could not be managed on a “batch” basis. To improve the sleep fragmentation of the on-call residents, it was suggested that non-emergent calls be delivered by text messages on a batch basis [18]. However, the pattern of unstable patient calls, as shown in this study, may disturb the sleep of the on-call residents all night long. Therefore, the protected sleep time is probably the best strategy to deal with this situation [15].

Early in the 1990s, Harvey pointed out that studying the night calls allowed specific strategies to reduce the number of calls to be identified [11]. However, no known interventions have been proposed to effectively reduce night calls in the following decades. The lack of front-line information may explain the knowledge gap in dealing with the night shift workload. That’s why we have attempted to perform the ground work on this issue to help provide some initial findings.

With regards to the methodology of the night call study, earlier studies tended to record data first-hand from nurses or doctors [10,11,14], while recent studies usually adopted the web system records [16,18,19]. The web system records can reduce the number of instances when data are missing, but might underestimate the events required to call if the responsible resident was already on the ward [19]. In our study, the night shift nurses were ordered to record every event in which they needed to call the physicians and they were, therefore, the ones to report the data first-hand. In addition, the web system records cannot help identify whether a call was placed by nurses or by doctors if it originated from the nurses’ station [11]. Therefore, the recording, as performed by nurses, provides valuable and correct information. A previous study demonstrated that nurses completed 95% of the records, while interns completed 86% of them [11]. The on-call residents may often be interrupted or they try to sleep at night, which may explain why they recorded the calls to a lesser extent than nurses. From this point of view, the nurses’ first-hand records appeared to be the most reliable ones. Further studies may combine nurses’ records with web registration data to make the design more accurate. In addition, it also worth recording the detailed managements done by residents after the calls. How patient care is organized and what resources are needed are essential for patient safety at night shifts.

Our study had several limitations that are worth noting. First, the reasons why patients called the night shift nurses were not studied. In a previous study, the reasons that made patients call nurses were different from the reasons that made nurses call doctors [20]. Our study, however, focused only on the need to call residents whose response and workload could therefore be studied. On-call residents may consult attending hospitalists, but this was not included on the record forms, either. Second, we only surveyed the needs for resident care at night, an aspect that could not be generalized to the day and evening shift. The care at night aims to provide the best possible sleeping environment for the patients [21]. The night call studies can reveal the patients’ real needs during the night, without being confounded by daytime activities. While most other studies were conducted at daytime, it is mandatory to gather the front-line information at night to improve nighttime problems [22]. Third, in our hospitalist service, we rarely have new admissions during night shifts. Therefore, the reasons for the calls were excluding the ones that were placed for new admissions. In other hospitalist programs, where night shift admissions are common, our data regarding the night workload may be underestimated. However, the calls for new admissions, if pooled into the statistical analysis, will inevitably distort the needs of inpatients during the night hours. Our study focused on inpatients’ calls at night, rather than on calls for managing admission process. Therefore, the previously admitted patients and new admitted patients should be considered separately. Forth, time spent on patient care should be an important endpoint in assessing physician workload, but we could not address it based on our data. Time spent on managing the patient’s call is highly related to the resident’s training and personality, which have been classified into “provider factors” in Horner’s framework and warranted further research. Fifth, the study was conducted in single academic medical center in Taiwan. The generalizability of our results to other settings has not been proved.

## Conclusion

The clinical state of the patients determines the workload during the night shift in a general medicine service. Both the cause and effect of the night calls differ among patients in different clinical states. Vital sign changes that needed assessment were the leading reasons for calls that were placed at night, and this was particularly the case for unstable and DNR patients. In the general medicine unit, DNR patients contributed substantially to the workload at night, to an extent comparable to unstable patients. Researcher should be reminded that beyond work hour, patient factors determine on-call workload. In different residency programs, factors other than work hour mandate investigation to allow fair comparisons. Further studies to predict the workload based on patient composition are warranted.
